# Using Novel Multimethod Evaluation Approaches to Understand Complex Food System Interventions: Insights from a Supply Chain Intervention Intended to Improve Nutrition

**DOI:** 10.1016/j.cdnut.2024.103776

**Published:** 2024-05-25

**Authors:** Mywish K Maredia, Eduardo Nakasone, Maria Porter, Stella Nordhagen, Vincenzina Caputo, Eric W Djimeu, Andrew D Jones, Mduduzi NN Mbuya, David L Ortega, Djeinam Toure, David Tschirley

**Affiliations:** 1 Department of Agricultural, Food, and Resource Economics, Michigan State University, East Lansing, MI, United States; 2 Department of Political Economy and Moral Science, University of Arizona, Tucson, AZ, United States; 3 Knowledge Leadership Team, Global Alliance for Improved Nutrition, Geneva, Switzerland; 4 Results for Development, Washington, DC, United States; 5 School of Public Health, University of Michigan, Ann Arbor, MI, United States; 6 Knowledge Leadership Team, Global Alliance for Improved Nutrition, Washington, DC, United States; 7 Africa Regional Office, Helen Keller International, Dakar, Senegal

**Keywords:** methods, project impact, complex interventions, supply chain, SMEs, Kenya, food environment

## Abstract

**Background:**

A “food system” approach to improve diet quality by intervening within areas such as food supply chains is gaining prominence. However, evidence of such interventions’ impact, and understanding of appropriate methods to evaluate them, is lacking.

**Objectives:**

We present an impact evaluation of an intervention that aimed to increase consumption of nutritious foods by supporting food-producing firms in Kenya. In doing so, we demonstrate how multiple methods, including those from other disciplines, can be used to evaluate a complex food systems intervention.

**Methods:**

Four methods focused on food-producing firms and their management, including a survey of intervention participants (*n* = 83 individuals), a “laboratory-in-the-field” experiment (*n* = 83 individuals), baseline/endline data on firm performance (*n* = 71 firms), and semistructured interviews (*n* = 19 firms). Three methods focused on consumers in neighborhoods targeted by a supported firm: a randomized field experiment tested effects of making a supported product exhaustively available on consumers’ purchases and consumption (*n* = 1295 consumers); 3 discrete choice experiments (*n* = 1295 consumers) tested factors influencing consumers’ willingness to pay for foods with relevant characteristics.

**Results:**

Among firms, we saw suggestive evidence of increased networking and business relationships, while laboratory-in-the-field experiments indicated the intervention might foster cooperation among participants. Qualitative interviews suggested that the intervention enabled firms to increase production, improve management, increase revenues, and lower costs. Baseline/endline data confirmed a positive effect only on the launch of new products and hiring workers. In the field experiment, consumption of the supported product increased in areas where it was made available relative to a control group, but this did not increase overall consumption of the food type or dietary diversity.

**Conclusions:**

Results showed positive signs of the intervention improving firm-level outcomes but limited impact on consumers’ diet quality. The evaluation also demonstrates how diverse methods can be used to evaluate complex interventions.

## Introduction

In recent years, the global food and nutrition community has increasingly advocated for a “food system” approach to improve diet quality (among other outcomes) by intervening within the processes related to the production, processing, distribution, preparation, and consumption of food [[1], [2], [3], [4], [5]]. This shift was made particularly clear at the 2021 UN Food Systems Summit (UNFSS) where over 110 countries submitted pathways that set out their country’s objectives and actions for transforming their food systems [6].

Food system interventions focused on nutrition have largely targeted the parts of the system most proximate to the consumer: the food supply chain and food environment [[7], [8], [9], [10], [11], [12]]. The food supply chain describes the processes involved in getting food from the farm to the consumer and encompasses food production, processing, distribution, and retail, while the food environment is “the interface that mediates people’s food acquisition and consumption within the wider food system” [13]. The food environment—the end of the food supply chain most proximate to consumers—affects food consumption through food availability, affordability, and desirability.

Interventions that work through the food supply chain and food environment have considerable appeal: even low-income households in rural areas of low-income and middle-income countries (LMICs) purchase much of their food [14,15], and these purchases can play an important role in improving diet quality [16,17]. The quality of diets thus emerges largely from food supply chains, making them potentially powerful leverage points.

Through decades of research, the nutrition community has built strong evidence on the impact of “traditional” nutrition interventions, such as supplementation [[18], [19], [20]]. Evidence on the impact of interventions that work through the food supply chain (including the food environment) is much sparser [11,[21], [22], [23]]. Although the UNFSS helped consolidate evidence on many food systems–related topics [24], there was limited evidence provided on effects of direct supply chain interventions on nutrition.

Obtaining more evidence on the topic is not straightforward. Evaluating these types of interventions is challenging, given their complexity [25,26]. Generating credible evidence requires a broad approach. This broader scope, coupled with the necessity that interventions adapt to changing circumstances over time, often limits the applicability of traditional evaluation methods, such as randomized trials: clear statistical evidence of causality will frequently be unattainable [27]. There is thus an urgent need to expand the evidence base on food system approaches to improve nutrition and to explore new methodologic approaches for doing so.

This article contributes to filling this gap by presenting an evaluation that combines several methods to understand the impact of a food systems intervention in Kenya. The intervention was designed to increase consumption of nutritious foods by supporting food-producing firms, with the expectation that this would generate measurable increases in the availability, affordability, or desirability of healthy foods. Owing to the abovementioned challenges, our evaluation did not focus on using a single method to evaluate the ultimate outcomes of the intervention (e.g., changes in diet). Instead, we aimed to reduce the complexity by separating the intervention into different sections with different outcomes and evaluating each in turn.

The aim of this article is 2-fold. First, we aimed to demonstrate how this multimethod, theory-of-change-focused approach (including both its advantages in achieving simplification and the limitations of doing so) can be used to evaluate a complex food system intervention. Such experimentation is important, as there are numerous donor-funded development programs and government initiatives that aim to improve nutrition through improved food availability—but little evidence to date on their impact. Second, we aimed to contribute to the body of evidence regarding the effectiveness of interventions that operate through food supply chains to impact diet quality and food choices. Along both of these lines, we believe that the article insights can help inform the work of program implementers and evaluators.

## Case Study Intervention

The case study intervention examined in this article is the marketplace for nutritious foods (MNF) project, implemented by the Global Alliance for Improved Nutrition (GAIN) in Kenya from 2018 to 2021. MNF aimed to support small-sized and medium-sized enterprises (SMEs) that produce and sell nutritious foods to expand their production and broaden the reach of their products among low-income populations. SMEs play critical roles in food systems worldwide, particularly in LMICs [28,29]. In addition to primary production [30], SMEs are also highly active in storage, distribution, wholesale, and processing. Altogether, traditional food supply chains, made up primarily of SMEs, constitute 50%–80% of the food economies in LMICs of Asia and Africa [31,32]. They have been argued to play a critical role in promoting consumption of nutritious foods among lower-income consumers [33]. SMEs in LMICs, however, face many systemic constraints. These include limited information (e.g., about market demand, production methods, and new technology), weak institutional capacities, and limited access to finance [[34], [35], [36], [37]].

MNF aimed to address some of these constraints among nutritious food-producing SMEs, namely those in the latter stages of the supply chain (processing, distribution, and retail), using them as conduits to increase the availability of nutritious foods to consumers (particularly those with low incomes). It had 2 components: the Community of Practice (CoP) and the impact accelerator (IA).

The CoP provided a platform to foster learning on topics related to business and nutrition as well as networking among entrepreneurs, businesses, civil society organizations, and public agencies through meetings, trainings, newsletters, and communications in online platforms. Training was intended to directly improve SME management practices, while stronger networks among participants were meant to increase mentorship and information sharing. These interventions were intended to make firms more efficient and competitive in the market, thereby improving their profitability and increasing sales of nutritious foods to consumers. Thus, the aim of these interventions was to increase the desirability, affordability, and availability of nutritious food in local markets targeting low-income consumers.

**FIGURE 1:**
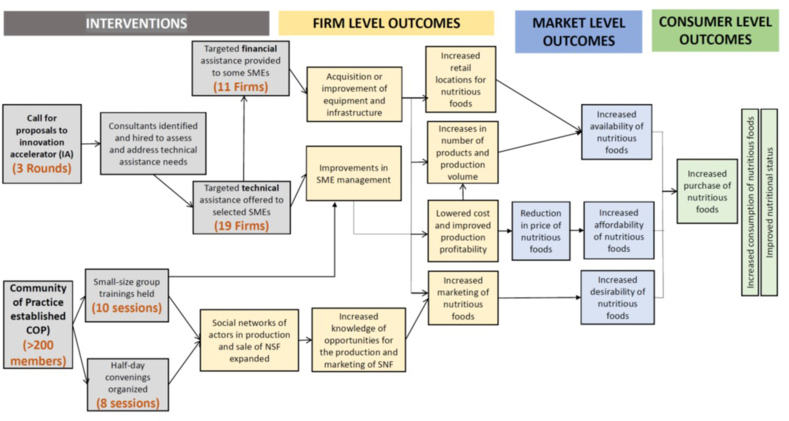
Impact pathway of the marketplace for nutritious foods project. CoP, community of practice; IA, innovation accelerator; SME, small-sized and medium-sized enterprise.

The IA provided technical and financial assistance to food-producing SMEs, which were selected through competitive calls for proposals. Proposals were evaluated by a committee based on the following 6 criteria: nutritional quality of the food produced, innovation, business capabilities, development objectives, feasibility, and likelihood of attracting further investment. All selected firms received technical assistance (TA), while the most promising also received a grant to help implement their business plans. TA was expected to improve management practices, production capacities, and marketing strategies, leading to greater and more efficient production better targeted at consumer markets with the greatest potential for expansion. Grants financed better equipment and infrastructure for firms, which could contribute to increased production and sales locations, lower costs, and improved marketing. The supposition was that this would in turn increase availability, affordability, and desirability of nutritious foods (i.e., impact characteristics of the food environment), potentially leading to greater purchase and consumption of these specific food products and perhaps these food categories more broadly. MNF’s impact pathway, depicting these hypothesized processes, is shown in Figure 1.

By the end of the intervention, over 200 SMEs were part of the CoP, and 8 half-day convenings and 10 targeted trainings had been conducted (numbers shown in Figure 1). Under the IA, 19 firms were selected for TA across the 3 rounds, and they received support on market research, business plan development, product development, and quality assurance. A subset of 11 firms also received grants (up to United States $90,000, with an average of United States $75,000) to purchase equipment, improve infrastructure, and increase production capacity.

Previousr studies have found mixed evidence on the effects of technical and financial assistance [[38], [39], [40], [41], [42], [43], [44], [45], [46]] and of business networks on SME performance [[47], [48], [49], [50]]. Very little research, however, has focused on nutritious foods or examined how such programs might affect consumers’ food access. The MNF evaluation thus sought to fill these gaps.

## Designing the Evaluation Methods

Several aspects of the MNF made it difficult to apply a standard evaluation approach. The complexity of the program meant that assistance to firms would need to filter through several causal steps before resulting in changes to food environments or impacting consumers. In addition, the 2-year timeframe of the evaluation was short relative to the long timeframe expected for achieving the targeted results. Moreover, the number of firms involved was very small in comparison with the overall market. Finally, the selection process was not random: participating firms either self-selected (i.e., in the CoP) or were selected by a committee that chose the most promising applications based on the above-described criteria (i.e., in IA).

**FIGURE 2:**
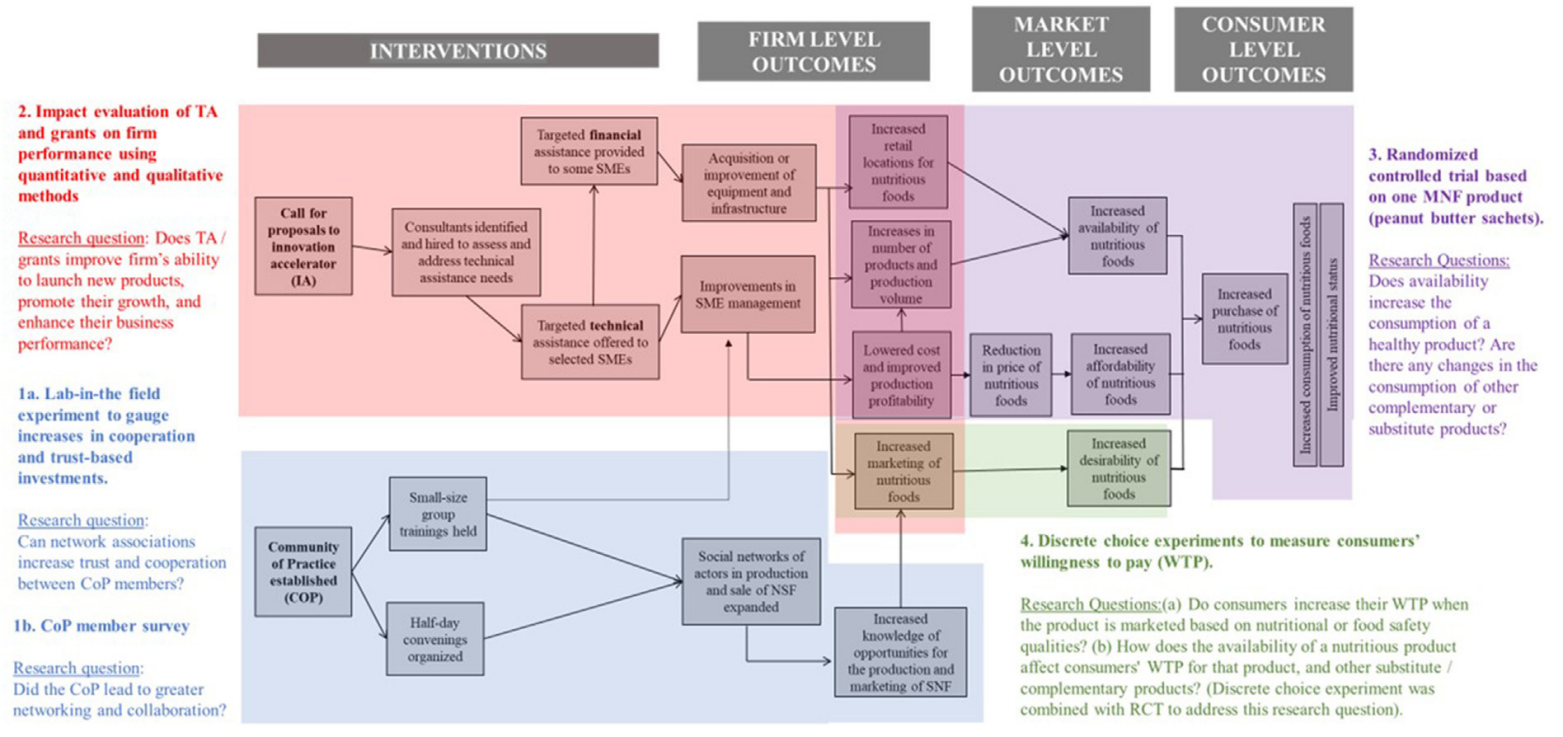
Components of the impact pathway analysis and methodology. CoP, community of practice; IA, innovation accelerator; MNF, marketplace for nutritious foods project; RCT, randomized controlled trial; SME, small-sized and medium-sized enterprise; TA, technical assistance: WTP, willingness to pay.

These characteristics, like challenges faced in many food systems interventions, were not conducive to a “traditional” impact evaluation approach, which typically examines final outcomes across an intervention and a comparison group or sequentially traces effects along the causal pathway. Instead, we drew on program theory [51] and designed an evaluation where we split the program’s impact pathway into several smaller components to assess different linkages within it and determine whether the different components contributed to the overall goal of the intervention. Owing to the diverse nature of these components, we used different methods to test each component (Figure 2).

In the following sections, we provide more detail on the methods and results for each study component, organized by the specific research question answered. All interactions with the study participants followed the Institutional Review Board guidelines of Michigan State University and the Maseno University Ethics Review Committee.

## Methods and Results, by Research Question

### Did the CoP increase networking, trust, and cooperation?

To examine whether the CoP achieved its aim of fostering networking and increasing information sharing and collaboration among firms, we used 2 methods (blue area in Figure 2). The first was a brief descriptive survey administered to CoP members at 5 CoP events, reaching a total of 83 unique members. (The survey was conducted at 5 CoP events organized by GAIN between August 2019 and January 2020 and does not capture the opinions/perceptions of past participants who dropped out or nonparticipating CoP members.) Survey findings suggested the CoP increased networking: 67% of respondents interacted with another CoP member socially or professionally outside of the CoP, on average, respondents interacted with ≥5 more people at the meeting whom they had not known beforehand, 39% reported that they had provided mentorship to another CoP member, 36% had sold or purchased from another CoP member firm, and ∼85% had learned a new management practice or production technique from another CoP member. ∼40% cited gaining new business and product development ideas through CoP membership, while 34% reported expanding their market. However, in the absence of a counterfactual group, the marginal contribution of the CoP (particularly to the networking-focused outcomes) was less clear, as 64% of respondents already belonged to another association promoting networking among firms, while 79% belonged to an entrepreneurs’ WhatsApp group. Given these other opportunities, participants in the convenings may have been able to establish collaborations with other firms even in the absence of the program. The survey was also subject to reporting bias and other weaknesses. However, it did provide support for the potential functioning of several steps in the impact pathway.

The second method focused on whether CoP participation increased trust among CoP members [52], a necessary step before sharing certain types of business information or entering into collaborations. We conducted an experimental economic game with CoP participants based on a previous study [53]. Each participant received an initial endowment of experimental currency (i.e., 10 tokens, each worth 100 KSH or about United States $1), and they were divided into 2 groups—senders and receivers. The sender was presented with an opportunity to increase their endowment through a joint investment by deciding an amount to send to the recipient. The recipient received triple this amount, then chose how much to return to the sender. Because of this multiplication effect, joint investments are collectively profitable, but the size of these collective gains depends on how much the sender decides to send, and its distribution depends on how much the receiver reciprocates. The game is meant to test trust: the sender’s initial decision on how much to send depends on their trust in the recipient, the recipient’s decision on how much of the profit to return to the sender depends on the recipient’s trustworthiness. We used 2 experimental treatments, where participants played with another anonymous CoP member or with another CoP member whose identity would later be disclosed. This allowed us to test whether potential personal relationships developed through the CoP increase trust. (The experiment also tested the role of uncertainty and imperfect contract enforcement, but those results are not discussed in this article in the interest of brevity. Please see [52] for a full discussion of results. A third experiment was originally included but not used in the final analysis.)

The results show that senders send more money in games where they knew that participants’ identities would be revealed. This suggests that personal relationships and norms do help enhance trust, and informal associations like the CoP can help promote cooperation between firms by offering them opportunities to build these relationships, as envisioned in the impact pathway. In the real-world, such trust could result in greater cooperation among firms, increasing their willingness to share technical/management practices, investment opportunities, and business ideas, or to enter into transactions or joint ventures.

### Do technical and financial assistance improve firm performance and the launch of new food products?

The second piece of the evaluation investigated the impact of the IA (red area in Figure 2). We examined whether firms that receive TA and grants are better able to launch new products, as well as whether they can reap the benefits of these new products to increase their staffing or product sales, profits, or assets. To address these research questions, we used mixed methods involving qualitative and quantitative approaches. Results of this evaluation piece are published previously [54].

In the quantitative analysis, we collected data from 19 firms that received TA from 3 rounds of IA proposals that took place between 2018 and early 2019; 11 of these firms also received a grant. We sought to build counterfactuals (ex-ante): for each “winning” firm, we identified between 1 and 5 control firms among the applicants that did not receive support but were similar to those that did, based on similar product and location and relatively high scores from the selection committee in the same application round. We conducted 2 survey rounds among treatment and control firms: a baseline before the firms had received assistance, and an endline about a year after the baseline. To these data we applied 2 statistical methods: *1*) ANCOVA regressions of firms at endline, including baseline covariates [55], and *2*) fixed-effects regressions that directly compared paired samples of treatment firms with their corresponding control firms.

The analysis showed that the IA was successful in achieving one of its main goals: to help SMEs launch new products. Our estimates suggested that firms that received TA (by itself or with grants) were ∼30 percentage-points more likely to introduce a new product. We also found suggestive evidence that beneficiary firms employed 7–15 more workers, although this effect waned at the onset of the COVID-19 pandemic. We found no quantitative evidence that the IA had an impact on other firm-level outcomes (sales, profits, or assets), but we cannot rule out the existence of impact due to the small sample and limited evaluation window.

We complemented this quantitative analysis with qualitative semistructured interviews with managerial staff (*n* = 28) at 19 firms (in the qualitative component, we interviewed firms that had received both technical assistance and grants), providing richer but more subjective information about the perceived impact of intervention support on firms’ performance and capacity to produce and market nutritious foods, from the perspective of the firm. In addition to those that received support in the 3 rounds covered by the quantitative analysis, we interviewed 8 firms that had received this aid in previous rounds, enabling us to explore longer-run effects. (Consistent with the quantitative sample in Nakasone et al. [54], the firms included in the qualitative portion of the evaluation included 11 firms from 3 rounds of the IA conducted between 2018 and early 2019. We decided to interview some firms that received both TA and FA in previous rounds of the IA to investigate potential longer-term impacts. This additional sample was not reported in the aforementioned study [54]). Interviews were audio-recorded and transcribed verbatim and, then, thematically coded and analyzed to detect common patterns.

The qualitative results show that interviewed firms perceived the impacts of the TA and grants as markedly positive. Firms indicated that the TA improved their understanding of market demand, their ability to design and enact business plans, and other production and management topics. Grants facilitated increases in the scale and capacity of firms to produce their products, helped them reduce input costs, employ more staff, improve safety standards, and diversify product lines. Along these lines, firms perceived that these changes led to increased sales and profit. Firms uniformly reported that the benefits of the TA and grants persisted, in some cases for several years.

Respondents from 6 of the 8 firms who had received assistance in previous MNF rounds to the main round under evaluation also identified “copycat” firms: firms that had begun production of a similar food product or started using a similar technology, sales model, or marketing approach as the respondent’s firm because of the respondent firm’s adoption of that product or approach following the assistance—suggesting an indirect effect of the intervention.

### Does increased availability of nutritious food increase its consumption?

The small size of MNF relative to the market in which it was operating, together with the short duration of the evaluation, limited our ability to detect changes in market-level or consumer-level outcomes along the intervention’s impact pathway (the purple area of Figure 2). We, therefore, aimed to experimentally imitate the best-case medium-term scenario for the intervention: support to a firm leading to wide availability of a new nutritious food in the firm’s area of operation. To do this, the research team made one MNF-supported product exhaustively “available” by distributing it to all retailers targeted by the firm in randomly assigned segments of the food environment in 5 of Nairobi’s low-income neighborhoods, and not in others. The experiment was performed for 12 weeks. In total, 477 retailers agreed to stock the product on a consignment basis. The product was sold at the standard market price of 20 KES each (approximately United States $0.18) and earned the retailer 5 KES profit per sachet. The detailed methods are reported previously [56].

The nutritious product chosen was peanut butter sold in small 40-g sachets, with advantages over existing homemade peanut butter products sold in low-income neighborhoods: the size and packaging made it affordable and convenient as a snack. It had no added sugar or oil, and its higher-quality production and packaging reduced risk of aflatoxin contamination [57], as indicated by a certification logo on the package from the Kenya Bureau of Standards. It was also cost-competitive with alternatives such as other formal sector peanut butter, homemade peanut butter, butter, and jam.

There were several reasons why we chose to focus on peanut butter sachets. (When designing the evaluation, we considered working with other intervention-supported products—e.g., fresh fish, leafy green vegetables, and bananas. Unfortunately, however, we would not have been able to trace whether consumers purchased these products from intervention-supported businesses or from other sources.). Peanut butter sachets are inexpensive, so even poor households are not likely to face significant cash constraints to purchase them. Households were already familiar with peanut butter, avoiding the barrier that households can be reluctant to try new foods. As the product is packaged and branded, it was easy to specifically identify and trace, which would not be the case for many other products sold in Kenyan markets (e.g., vegetables, fruit, eggs, and most meat and fish). Another benefit of its packaging was that we could be sure about the product’s nutritional benefits and that it was processed under high-safety standards. Finally, the safety of this product was certified by the Kenyan Bureau of Standards, which can be an important benefit for customers [58].

A subsample of shops in the treatment and control groups were targeted for a survey, sales tracking, and product availability checks. Responses from these shops confirmed the approach functioned as planned: among treated retailers, 91% sold the product (wide availability), while only 3% did so in the control group (almost no availability). We also recruited regular shoppers at these stores for a household survey on consumption patterns. The baseline survey was conducted in October 2019–March 2020 and the endline in March–July 2021, with a total sample of 1295 consumers.

The results showed that making the nutritious product more widely available increased its purchase and consumption by households from both treatment and control segments, but the increase was significantly larger among consumers in treatment segments. In the 6 mo before the endline survey, 97% more consumers from the treatment segments consumed the product than consumers recruited from control segments (16.3% of consumers compared with 8.3%), and consumers who shopped in treatment segments purchased 3 times the quantity on average as those in control segments. However, the availability of this 1 brand of peanut butter did not increase overall consumption of peanut butter as a food, as these increases were offset by declines in consumption of other types of peanut butter. Consumers in the treatment group consumed less peanut butter sold by formal sector brands with not only similar food safety standards but also with added sugar or fat, suggesting a small potential improvement to diet quality.

### Does greater availability of a nutritious food increase willingness to pay for it? Are consumers willing to pay more for nutritious or safe products?

Success of a market-based nutrition intervention is dependent on a successful business case—that is, the SMEs involved being able to earn sustained profits from selling the nutritious food product. The intervention’s impact pathway assumed that support to nutritious food-producing SMEs would help them expand to additional retail areas and increase marketing of their products, expected to drive greater consumer demand—and thus sales and revenues for the firms. This increased desirability and demand for nutritious products can also impact consumption of other foods.

To test this (green area of Figure 2), we coupled our endline household survey in the randomized controlled trial (RCT) (described in the previous section) with 3 hypothetical choice experiments with the 1295 consumers in our sample. The first included 3 product alternatives (peanut butter, margarine, and jam), each offered at 3 hypothetical price levels: 15, 20, and 25 KES. In each of 8 choice questions, the respondent could choose among 3 product alternatives offered at different prices or to not purchase any. This allowed us to capture changes in the consumption of related products (e.g., margarine and jam) when a nutritious product is launched. As the choice experiment was embedded within the consumer RCT, we were able to assess whether households were willing to pay more for the nutritious product in areas where it was made widely available. Results showed that consumers had a strong preference for peanut butter compared with jam, but not more than margarine, a product that directly competes with peanut butter. There was no significant difference in preferences for peanut butter between consumers from treatment and control segments, suggesting that expanding product availability did not increase desirability of peanut butter over the less nutritious option, margarine.

The second choice experiment conducted on a subset of consumers, involved selecting between different pairs of peanut butter sachets, which included either information about high protein content, a message indicating it was aflatoxin safe, or no nutritional or safety claims. This echoed the characteristics of the product used in our RCT. The products were offered at 4 price levels: 15, 20, 25, and 30 KES. Similar to the previous experiment, in each of the 8 choice questions, respondents were presented with 3 product alternatives offered at different prices to choose from or they had the option not to make a purchase. This allowed us to investigate whether nutritional information and safety claims can help increase consumption of new safer and more nutritious products among lower-income households. The results showed that respondents valued products bearing nutritional and food safety attributes more than unlabeled products, which were chosen by <20% of respondents. The “Aflatoxin Tested” label was preferred over the “High Protein” label, especially when purchasing for children or the family. These findings suggest that labels reporting health and safety benefits help increase demand and willingness to pay.

These results are consistent with the third choice experiment, which was conducted using another intervention -supported product—enriched yogurt—on another subset of the consumer sample. The full method is described elsewhere [59]. That experiment found that marketing strategies and informational campaigns that effectively link the health benefits of product enrichment, such as vitamin-enriched and nutrient-enriched yogurt, to consumer health can significantly boost the product’s appeal and the willingness of consumers to pay a higher price. Particularly, when these campaigns are directed toward women and households with children, they show a substantial increase in the demand for these healthier yogurt options in the experimental setting, underscoring the importance of messaging in influencing consumer behavior in these demographic groups. Both the second and third choice experiments confirm a potential role for SMEs in addressing micronutrient deficiencies in vulnerable populations through product innovation and targeted marketing.

## Discussion: Piecing the Puzzle Together

This study had 2 aims: *1*) demonstrating how multiple methods can be used jointly to evaluate a complex food systems intervention and *2*) contributing to the evidence on the effectiveness of food systems interventions that work through food supply chains to impact diet quality. We consider each of these in turn in this section, followed by recommendations for improvement in the future.

### Using multiple and diverse methods to design a complex impact evaluation

Like many complex food systems interventions, this impact evaluation faced several challenges: the many-step impact pathway, the small size of the intervention relative to the market/population, and the short duration of the evaluation period. These all limited our ability to use standard impact evaluation approaches at the food environment or consumer-level.

To address these limitations, we separated the intervention’s impact pathway into distinct components and applied different methods to evaluate each of them. These methods, and their strengths and limitations, are summarized in Table 1. This enabled us to adapt to the different characteristics of the intervention components and their varying objectives and timing. In particular, 1 area where we benefited strongly from interdisciplinary collaboration was in using the laboratory-in-the-field and choice experiment methods (primarily drawn from economics). These allowed us to test concepts that would be hard to capture in observational data, as we were able to alter specific parameters and observe how participants change their decisions in response, without confounders. Although these have limitations (namely the choice experiments being hypothetical and both being in non-real-world settings and thus harder to generalize from), they were highly informative, given the time and budget limitations of the study.

**TABLE 1:** Summary of research questions, methods, strengths, weaknesses, and results

Research question	Methods used	Method strengths	Method weaknesses	Results
Did the CoP increase networking, trust, and cooperation?	CoP member survey	• Rapid, simple • Enabled adding additional elements to impact pathway, based on participants’ own perceptions	• Self-reported data only and subject to reporting bias • Only short term • No counterfactual	CoP members reported interactions among members, mentorship, and learning, and in some cases business relationships. Additional benefit compared with other networks unclear
	Laboratory-in-the-field experiment with CoP participants	• Experimental rigor • Incentives provided to encourage realistic decisions • Tests a hard-to-measure concept: trust	• Somewhat resource intensive, including the need for researcher expertise • Cannot test whether CoP led effectively to actual business collaborations	CoP might be effective at fostering cooperation and trust-based investments among participants
Do technical and financial assistance improve firm performance and ability to launch new food products?	Quantitative analysis of baseline and endline data of firms receiving TA, compared with matched firms that did not receive TA	• Captured key business data of high relevance • Enabled comparison to a matched group to simulate an experimental approach	• Small sample and short evaluation window limited explanatory power of analysis	Firms that received support were more likely to launch new products and hire more workers, but no measurable impact on sales or assets
	Semistructured interviews with firms that received grant assistance—11 firms from the most recent 3 rounds and 8 firms from previous rounds ≤5–6 y before	• Centers on participants’ perceptions • Possible to use without baseline data, enabling studying a longer period • Enables identifying unintended consequences as perceived by participants • Facilitates interpretation and contextualization of quantitative results	• More subjective information • No ability to estimate effect size or CIs • No comparison group/counterfactual	Firms increased their production of nutritious products, improved their management practices, achieved larger revenues, and/or lowered their production costs
Does increased availability of a nutritious food increase its consumption?	Field experiment making a supported product available exhaustively in a random subset of retail stores, with baseline and endline data on consumers that shopped at treated and control stores	• Experimental, isolating effect of increased availability alone • Real-world setting • Enabled examining effects on comparable and substitute products and diet quality	• Resource intensive • Requires careful supervision of the implementation’s adherence to experimental protocol • Required collaboration of the firm and retailers	Consumption of the product increased in areas where it was made available relative to a control group, but this did not increase overall consumption of peanut butter, impact the consumption of potential substitute products, or affect household dietary diversity. There may have been some diet quality improvement due to switching away from products with added sugar or fat
Does greater availability of a nutritious food increase willingness to pay (WTP) for it or affect WTP for other substitute/complementary products?	Discrete choice experiments (coupled with RCT)	• Allows for controlled examination of how different parameters influence consumers’ choices • Relatively simple to implement	• Hypothetical	No significant differences in WTP for peanut butter and other less nutritious substitute/complementary products between areas of wide availability and control areas, suggesting that availability of a nutritious product does not lead to higher product desirability or lower desirability of less nutritious products
Are consumers willing to pay more for nutritious or safe products?	Discrete choice experiments with products of 2 SMEs supported under IA•		• Hypothetical	Both nutritional and food safety information increased consumers’ WTP for the peanut butter product and health and nutrition-related information increased consumers’ WTP for enriched yogurt, suggesting that nutrition-related, health-related or food safety-related marketing and labeling can help increase demand

Abbreviations: CoP, community of practice; IA, innovation accelerator; RCT, randomized controlled trial; SME, small-sized and medium-sized enterprise; TA, technical assistance; WTP, willingness to pay.

Moreover, the randomized field experiment of supplying peanut butter to randomly selected market segments enabled us to effectively take an evaluation shortcut: to create a hypothetical situation envisioned within the impact pathway but not actually achievable within the intervention timeframe. Its random assignment made it possible to attribute the observed change (purchasing) to the intervention (product availability). Previously, few randomized experimental approaches have been used to estimate the impact of altering food availability on consumption [60]. However, this type of nonconventional thinking is essential when considering how to make feasible impact evaluation approaches that would normally take years to conduct.

Finally, we applied qualitative methods to mitigate the small sample size, provide flexibility, and enable inclusion of a broad set of research questions [61]. For example, qualitative interviews made it feasible to capture the indirect effect of nonsupported firms imitating products or business approaches of the intervention-supported firms, resulting in potentially larger effects of the intervention on the market than anticipated.

One evaluation challenge that we did not manage to surmount was the relatively small sample size of SMEs. The small sample size entailed a need for a very large effect for it to be statistically significant, at the same time, the short intervention timeframe provided less time for such an effect to be generated. (Power calculations based on baseline data suggest that, to detect a statistically significant impact, SMEs would have needed to increase their typical monthly sales by ∼80% or increase their profits by 110%. These magnitudes are implausible, particularly given the timeline of our evaluation.) Sample size constraints are neither unique to food systems interventions nor universal among them, but they are more likely to be a serious challenge with food systems interventions, as the unit of analysis is often at a higher scale than an individual or household—such as a company, market, food environment, or value chain.

### Did or could the intervention lead to improved diet quality?

The philosophy behind our evaluation design was that, by considering an intervention that was designed based on a clear impact pathway and rigorously assessing key parts of that pathway, we could make an informed judgment about the likely overall effect of the intervention, even if directly assessing that effect was not possible.

Moving from left to right across the impact pathway (Figure 1), we find moderately strong support for its first few steps. Survey evidence suggested that CoP participation led to interactions among members and learning, and in some cases business relationships, while laboratory-in-the-field experiments suggested the CoP might be effective at fostering cooperation and trust among participants. Qualitative interviews with firms suggested that intervention support made them better able to increase their production of nutritious products, improve their management practices, achieve larger revenues, and lower their production costs. More rigorous quantitative baseline/endline data confirmed a positive effect only on launch of new products and (to some extent) hiring more workers, but no impacts on sales, profits, or assets—although it was also underpowered to do so over the short time and given the small sample size. Taken together, these results suggest that the CoP, TA, and grants were effective tools to support SMEs to produce nutritious foods.

However, the intervention does not seem to have been as effective at improving market-level and consumer-level outcomes. In the RCT, consumption of the product increased in areas where it was made available relative to a control group, but this did not increase overall consumption of peanut butter or impact the consumption of potential substitute products (butter or jam), nor did it affect household dietary diversity. There may have been some diet quality improvement due to switching away from products with added sugar or fat. Previous research has generally found positive associations between product availability and consumption, but with some variation [11,12,62,63]. Associated choice experiments suggested that availability did not increase the product’s desirability.

Taken together, the results suggest meaningful positive impacts on SMEs but (albeit more speculatively) only limited likelihood of impacting the healthiness of consumer diets, the main objective of the intervention. At the same time, the RCT involved only 1 product supported by the intervention (peanut butter in small sachets), it may not be representative of all diverse food types included in this intervention, or of efforts to improve food availability more broadly. Assessing a wider range, and different types, of products could perhaps have led to more robust conclusions. However, feasibility of doing so may be limited by the challenge of tracing many types of nutritious foods (e.g., vegetables, fruit, and eggs). Without being able to clearly connect a given product to a given company, a change in purchase or consumption of that product cannot necessarily be connected to its up-chain source or intervention. [A similar challenge was noted in a recent RCT on fruit and vegetable consumption in Vietnam, which could not separate purchased fruit and vegetables (more closely connected to the intervention) from those consumed from own production] [60].

### Recommendations for future interventions and food systems for nutrition evaluations

Considering the scale of MNF compared with the market in which it operated (a scale that was comparable with other market-based approaches), we conclude that such an approach is unlikely to generate market-level and consumer-level effects that are large enough to detect statistically. That does not necessarily mean that those effects are not meaningful for those who experience them, but it does indicate that larger-scale and more systemic efforts are needed. We have 5 suggestions for what these may be.

First, larger financing interventions (such as blended finance-based impact investing) [[64], [65], [66]] could work to enhance access to finance at a higher level and for a greater number of food-producing firms at various points of the supply chain [67], increasing the amplitude of the market-level effects beyond what is possible through a grant-based approach like MNF. Second, whole-of-supply chain approaches (i.e., those that work with actors along a supply chain from input provision up to retail, as opposed to just one supply chain actor) may have greater opportunity for impact, particularly where there is a bottleneck or weakness in the supply chain, such as an inability to source safe inputs due to poor food safety capacity at the production or handling stage [68].

Third, as availability of a nutritious product by itself did not seem to be an effective tool to promote increased consumption and thus healthier diets, it may need to be coupled with demand-side interventions, including targeted marketing campaigns to influence desirability and willingness to pay for healthier foods [[69], [70], [71]]. Our choice experiments provided support for such an approach by indicating that both nutritional and food safety information increased consumers’ willingness to pay for a product, suggesting that nutrition-relates or food safety-related marketing and labeling can help increase demand. Interestingly, only a small percentage of consumers of the peanut butter in the RCT cited its nutritional value or high quality as a reason for consumption, indicating considerable scope for increasing awareness of these potential purchase drivers. Even within the consumer-focused marketing space, a multipronged approach will likely be needed. Interventions with multiple information, education, and communication components tend to be more likely to have an impact on changing food consumption than simple informational interventions [72].

Fourth, to effectively improve the diets of the lowest-income consumers, marketing campaigns will be insufficient: nutritious foods will remain out of their reach without monetary support to purchase these foods. As such, subsidies on nutritious foods or social protection programs that can boost purchasing power are also needed. Although the MNF impact pathway initially envisioned potential improvements in food affordability as a result of the intervention, changes to food prices are very challenging to bring about without marketwide or societywide interventions. However, some increase in affordability could potentially be achieved in the future by supporting specific product adaptations (such as use of lower-cost ingredients or eliminating expensive packaging), which can increase affordability of nutritious foods among lower-income consumers [73].

Finally, there is a need for food system policies that reduce the cost of doing business, such as strengthened contract enforcement, increase access to credit, such as collateral registries [74,75], address supply chain bottlenecks that constrain growth or increase prices, and provide clearer information to consumers on which foods are and are not nutritious, such as front-of-pack labeling [76]. In practice, multiple changes on both the supply and demand sides, as well as in the enabling environment that support them, will be needed to unlock widespread increased consumption of nutritious foods.

Regarding recommendations for evaluation design, although all methods used had strengths, we highlight 2, laboratory-in-the-field and choice experiments from behavioral economics, which are relatively unused in nutrition research, with exceptions [[77], [78], [79]], but helped manage the complexity of this evaluation. They may prove to be more widely useful in food systems for nutrition evaluations to examine people’s food choices under controlled conditions. In contrast, qualitative methods, although widely used in international nutrition research [11,80,81], are not as common or as rigorously applied in the field of most of this article’s coauthors (economics), although they are growing in use. They provided some insight in this study, and with greater use of qualitative methods more systematically and rigorously applied for each evaluated component, we may have been able to better triangulate results and understand the deviations from the expected results—for example, why consumers chose not to consume more peanut butter in response to increased availability but rather replaced existing peanut butter purchases with the newly provided brand.

Significant opportunities exist to use diverse and innovative approaches to evaluate complex interventions such as this one. For example, simulation or modeling methods could be used to investigate how to apply economic models of a firm and a market to predict shifts in firm-level outputs and their subsequent impact on market-level parameters, taking into account the size and concentration of the market. Moreover, we were not able to assess how increased market-level affordability or desirability would impact consumer purchases. Existing research [[82], [83], [84], [85]] suggests a link between affordability and food consumption, but evidence on the topic from LMICs is limited [11,23]. These question could be examined through future choice experiments or, resources permitting, field experiments.

Finally, 1 of the key challenges of a multistudy, multimethod evaluation such as this is determining what is that overall “result.” We interpreted our results based on the program design theory and our understanding of the methods’ relative strengths and weaknesses, but as evaluations grow increasingly more complex under food system approaches, we recommend that better guidance and best practices for synthesizing results across diverse mixed methods evaluations be developed.

In conclusion, amid increasing attention to food systems approaches that aim to improve nutrition by altering characteristics of the food supply chain or food environment, there is a need to build evidence on which of these interventions work—and methods for doing so. Several recent articles have highlighted the importance of markets and supply chains in improving nutrition [15], the need for greater understanding of how to leverage supply chains to improve access and availability of nutritious foods [86], and why more innovative thinking is needed on evaluating food systems approaches to improving nutrition, particularly in LMICs [23,27].

This study contributes to filling these gaps by presenting an impact evaluation of an intervention that aimed to increase consumption of nutritious foods by supporting food-producing firms in Kenya, with the expectation that this would shift aspects of the local food environment. We show how multiple and diverse methods can be used to evaluate a complex food system intervention. Our results revealed some positive signs of the intervention improving firm-level outcomes but limited impact on consumers’ diet quality. To achieve the desired consumer-level outcomes, it is important to also consider demand-side interventions such as health and nutrition marketing campaigns and product labeling, as well as ways to alter prices to favor nutritious foods, for example, ‘true cost of food’-based pricing [87], which could help increase consumption of nutritious foods. We believe that the potential for improving nutrition through the food system remains strong. There is a need for more ambitious experimentation with both ways to do so and innovative research methods to understand their impacts, including those that cut across disciplinary boundaries and use multiple quantitative and qualitative approaches, based on primary and/or simulated data.

## Author contributions

The authors’ responsibilities were as follows – EN, MKM, D Tschirley, MP, ADJ, DLO, VC: led the evaluation discussed in the article; D Toure, EWD: provided input into design of the evaluation; SN, MNNM: provided feedback on the evaluation results; SN: wrote the article based on an internal manuscript by EN, MKM, D Tschirley; and all authors: read and approved the final manuscript.

## Conflict of interest

The authors report no conflicts of interest.

## Funding

This work was part of the Making Markets Work to Improve the Consumption of Safe and Nutritious Foods program, which was supported by Bill & Melinda Gates Foundation, Federal Ministry for Economic Cooperation and Development (Germany), International Development Research Center (Canada), Irish Aid, Ministry of Foreign Affairs of the Netherlands, and the Swiss Agency for Development and Cooperation. The supporting sources had no involvement or restrictions regarding publication.

## Data availability

Data described in the manuscript, code book, and analytic code will be made available on request pending application and approval.
